# Plasma concentrations of vitamin A and E and risk of dysglycemia in first-trimester pregnant Saudi women

**DOI:** 10.1186/s13098-020-00525-3

**Published:** 2020-02-18

**Authors:** Hebah Alawi Kutbi, Sahar Ali Hammouda

**Affiliations:** 1 Clinical Nutrition Department, Faculty of Applied Medical Sciences, King Abdulaziz University, 80215, Jeddah, 21589 Kingdom of Saudi Arabia; 2 Clinical Nutrition Department, College of Applied Medical Sciences, Taibah University, Al-Madinah Al-Monawarah, Kingdom of Saudi Arabia

**Keywords:** Vitamin A, Vitamin E, Antioxidant, Glycemic control

## Abstract

**Background:**

Existing evidence suggest that low concentrations of vitamin A and E may have a contribution to the development of diabetes complications; however, data regarding the status of vitamin A and E among individuals with prediabetes are lacking. This study aimed to examine the association of plasma concentrations of vitamin A and E with the glycemic control status among first trimester pregnant Saudi women.

**Methods:**

In this cross-sectional study, 1102 first trimester pregnant Saudi women were recruited from antenatal clinics. Sociodemographic and anthropometric information were collected, and laboratory analyses of blood glycated hemoglobin (A1C) and plasma vitamins A and E were performed. Subjects were classified as normoglycemic, prediabetic, or undiagnosed diabetic. Multinomial regression models adjusted for age estimated the adjusted odds ratios (aORs) and [95% confidence intervals (CIs)].

**Results:**

Among the sample, 78.8% (n = 868) had normal glycemic control, while 19.1% (n = 211) had prediabetes and 2.1% (n = 23) had undiagnosed diabetes. Plasma concentrations of vitamin A and E of prediabetic participants were at a level midway between that of normoglycemic and diabetic participants (p < 0.01). Compared to subjects with normoglycemic status, those with higher concentrations of vitamin A and E had lower odds of being prediabetic (aOR = 0.27 [0.21–0.35] and aOR = 0.95 [0.94–0.96], respectively) or diabetic (aOR = 0.18 [0.13–0.24] and aOR = 0.93 [0.92–0.94], respectively).

**Conclusions:**

Our findings indicate a possible contribution of vitamins A and E to the progression of prediabetes to diabetes. Future longitudinal studies are needed to elucidate the association between the antioxidant status and dysglycemia. Clinicians should monitor the glycemic and the antioxidant status closely and provide dietary guidance where needed.

## Background

Normal pregnancies are characterized by a physiologic resistance to insulin action and increases progressively to term. The glucose transports across the placenta to ensure adequate development and growth of the fetus; and as the pregnancy approaches the second trimester, insulin resistance becomes more pronounced [1]. Glycated hemoglobin (A1C) is a validated antenatal screening test for unrecognized type-2 diabetes mellitus (T2DM) and prediabetes in early pregnancy [2]. Historically, a diagnosis of gestational diabetes mellitus (GDM) was indicated for any diabetes during pregnancy. According to the current guidelines of the American Diabetes Association, the diagnosis of diabetes in the first to early second trimester is considered pregestational diabetes mellitus (Pre-GDM) [2]. In Saudi Arabia, the rising trends of obesity have led to greater incidence of T2DM, with greater proportions of pregnant women with undiagnosed T2DM [3]. A cohort study from Riyadh, Saudi Arabia, estimated the prevalence of Pre-GDM and GDM to be around 4.3% and 24.2%, respectively, indicating a great burden of diabetes among pregnant women in Saudi Arabia [4].

Pregnancy is a period of specific nutritional needs to support health of the mother and the fetus [5]. Vitamin A and E are micronutrients needed to detoxify free radicals. Any alterations in the status of vitamin A and E would be indicative to oxidative stress [6]. In pregnant women, insufficient intakes of vitamin A, poor bioavailability of provitamin A sources, periods of infection, and GDM are factors that have been previously linked to vitamin A deficiency [7, 8]. Several animal studies have provided evidence that vitamin A deficiency during pregnancy may result in adult metabolic diseases in rats through the effect on fetal islet development and subsequent islet function later in adulthood, which may suggest a possible role of vitamin A deficiency in the pathogenesis of diabetes in humans [9, 10].

The role of vitamin E as an antioxidant magnifies its importance in preventing phospholipid oxidation by harmful free radicals. In epidemiological studies, abnormal vitamin E concentrations have been linked to diabetes mellitus [6]. The suboptimal concentrations of antioxidant vitamins in diabetic individuals may induce oxidative damage and diabetes complications [11], while some studies suggested that the state of hyperglycemia accelerates production of free radicals via glucose autoxidation and protein glycosylation [12]. Given that little work was done investigating the antioxidant status of prediabetic and diabetic Saudi individuals, we aimed to assess plasma concentrations of vitamin A and vitamin E and examine the associations with the glycemic control status among first-trimester pregnant Saudi women.

## Subjects and methods

**Fig. 1:**
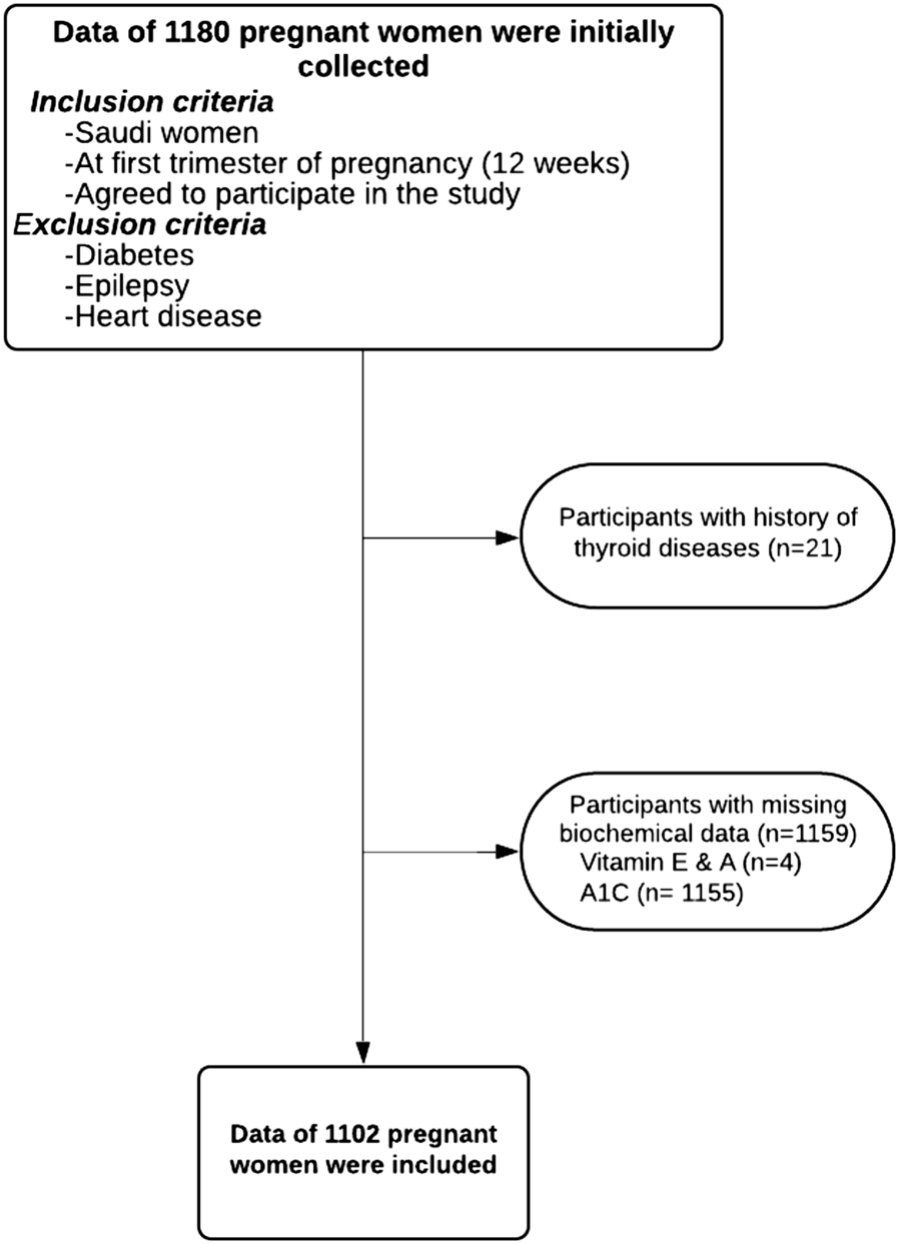
Flowchart of participant selection

The data were part of a cohort study collected between May 2009 and January 2011, wherein data of 1180 Saudi pregnant women were initially collected from several antenatal clinics located all around Al-Madinah Al-Monawarah city, Saudi Arabia [13]. Information on participants’ medical history were collected; and examination and laboratory analyses (plasma vitamin A, vitamin E, and A1C) were performed. The inclusion criteria include Saudi women at their first trimester of gestation (12-weeks pregnancy) who agreed to participate in the study. The exclusion criteria include those with a history of diabetes, epilepsy, or heart diseases. Data of participants with a history of thyroid diseases, or with missing biochemical data were further excluded. The final analysis included data of 1102 participants (93.4%) (Fig. 1). Approval of the study protocol was granted from the Ethical Committee of King Abdulaziz City for Science and Technology (KACST), (AT-28-113). A signed consent was collected from each participant in order to be included in this study.

### Sociodemographic and anthropometric variables

Information regarding participants’ age and education (less than a college degree vs. college degree or higher) were collected. The participants also reported their height and pregestational weight; and the body-mass-index (BMI) was calculated [14].

### Laboratory analyses

Fasting blood samples were collected from each participant, and the plasma was separated and divided into 8 aliquots and frozen at − 80 °C until the time of analysis. Blood A1C concentrations were assessed at the laboratory of Taibah University. Plasma retinol (vitamin A) and vitamin E (α-tocopherol) were measured by reverse phase HPLC. Using an Ultraviolet detector, vitamins A and E were detected at 325 nm and 292 nm, respectively. The diagnostic criteria of the American Diabetes Association was used to determine the glycemic control status of the subjects: normoglycemic if A1C < 5.7%, prediabetic if A1C ranged between 5.7 to < 6.5%, and undiagnosed type-2 diabetics if A1C ≥ 6.5% [2].

### Statistical analysis

The sample size of this study was determined based on a two-sided test, alpha of 0.01, power of 90%, and effect size of 0.10. Descriptive analyses were performed to present sample characteristics. Differences across subjects with normal A1C, prediabetes, and undiagnosed diabetes were tested using Kruskal–Wallis and Chi square tests. Differences in mean plasma concentrations of vitamin A and E according to the educational status of the participants were tested using the Mann–Whitney test. Spearman’s Rho test was used to evaluate the correlation between plasma concentrations of vitamin A and E with participants’ age, and to examine the correlations between A1C, vitamin A, and vitamin E at the different A1C concentrations. The linear associations between blood A1C and plasma vitamin A and E concentrations were examined using the linear regression analysis and associated 95% confidence intervals (CIs). The associations of vitamin A and vitamin E with A1C in subjects with prediabetes and undiagnosed diabetes were tested using separate multinomial regression analysis and associated 95% CIs, wherein normal A1C level was set as a reference category. The regression models were adjusted for subjects’ age, and adjusted odds ratios (aOR) were reported. Two-sided tests were used for all the analyses and a *P* < 0.05 was set to indicate significance. The data were analyzed using IBM SPSS (V.24).

## Results

**Fig. 2:**
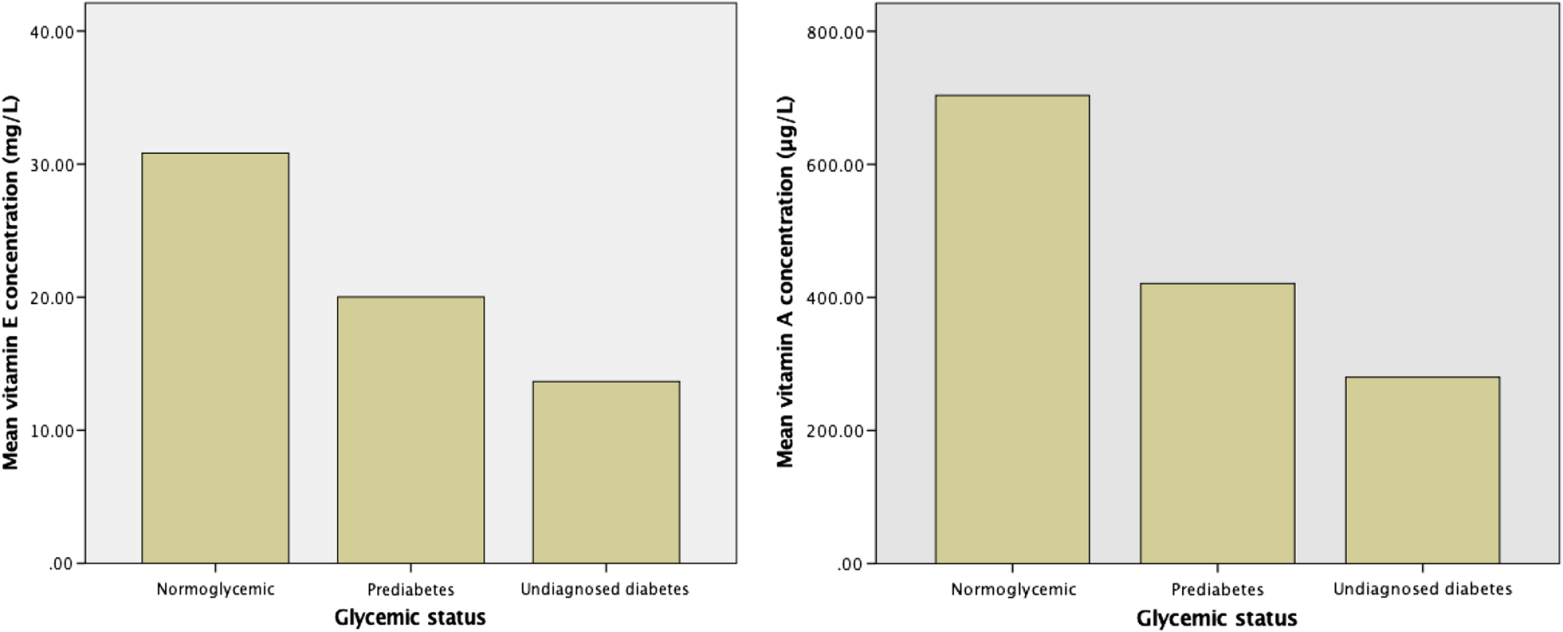
Mean plasma concentrations of vitamin A and E according to the glycemic status (n = 1102)

Descriptive statistics of the sample are illustrated as means ± standard deviations (SDs) and percentages in Table 1. The mean age and BMI of the participants were 28.0 ± 6.23 years and 25.3 ± 5.42 kg/m^2^, respectively. The majority of participants had less than college degree (n = 797, 72.3%). About three-quarters of the participants had normal glycemic control (n = 868, 78.8%), 211 participants (19.1%) were identified to have prediabetes, and 23 participants (2.1%) had undiagnosed diabetes. The mean A1C concentration of the sample was 5.19 (0.56). Means of vitamin A and E were 640.5 ± 155.0 µg/L and 28.4 ± 6.01 mg/L, respectively. Statistically significant differences in means of blood A1C and plasma vitamin A and E concentrations were observed among the participants with different glycemic status (Fig. 2).

**Table 1:** Characteristics of first trimester pregnant Saudi women according to the glycaemic status^a^ (n = 1102)

Variable	Normoglycemic control (n = 868)	Prediabetes (n = 211)	Undiagnosed type-2 diabetes (n = 23)	*P* values
Age	27.9 ± 6.25	27.9 ± 6.07	30.2 ± 6.78	0.239
Education, n (%)				
Less than college degree	635 (79.7%)	146 (18.3%)	16 (2.00%)	0.491
College degree or higher	233 (76.4%)	65 (21.3%)		
Pregestational BMI	25.2 ± 5.40	26.0 ± 5.39	26.4 ± 6.21	0.061
A1C %	4.97 ± 0.32	5.94 ± 0.19	7.03 ± 0.62	<.0001
Vitamin E (mg/L)	30.8 ± 3.81	20.0 ± 2.71	13.7 ± 5.93	<.0001
Vitamin A (µg/L)	703.5 ± 99.7	420.9 ± 61.7	280.00 ± 140.2	<.0001

All numbers are presented as mean ± standard deviation unless otherwise specified

*BMI* body-mass-index, *A1C* glycated hemoglobulin A1c

^a^Kruskal–Wallis and Chi square tests were used

Plasma concentrations of vitamin A and E were not found to be correlated with participants’ age (r = − 0.030 and r = − 043, respectively, *P* > 0.05). The mean plasma concentration of vitamin A did not statistically differ between that of lower education participants (646.3 ± 152.9 µg/L) and those with higher educational status (625.4 ± 159.6 µg/L), (*P* = 0.05). Similarly, mean plasma concentration of vitamin E among participants with lower education (28.6 ± 5.90 mg/L) did not statistically differ than that of higher education participants (27.9 ± 6.28 mg/L), *P* > 0.05). The plasma concentrations of vitamin A and E were strongly positively correlated (r = 0.979, *P *< 0.01) (Table 2).

**Table 2:** Spearman’s correlation of A1C, vitamin A, and vitamin E according to the glycemic status of 1st trimester Saudi women

Biochemical parameter	A1C < 5.7% (n = 868)	A1C ≥ 5.7% (n = 234)	Total (n = 1102)
A1C % * Vitamin E	− 0.964^a^	− 0.711^a^	− 0.976^a^
A1C %* Vitamin A	− 0.963^a^	− 0.809^a^	− 0.976^a^
Vitamin E * Vitamin A	0.964^a^	0.776^a^	0.979^a^

*A1C* glycated hemoglobulin A1c

^a^Correlation is significant at the 0.01 level (2-tailed)

**Fig. 3:**
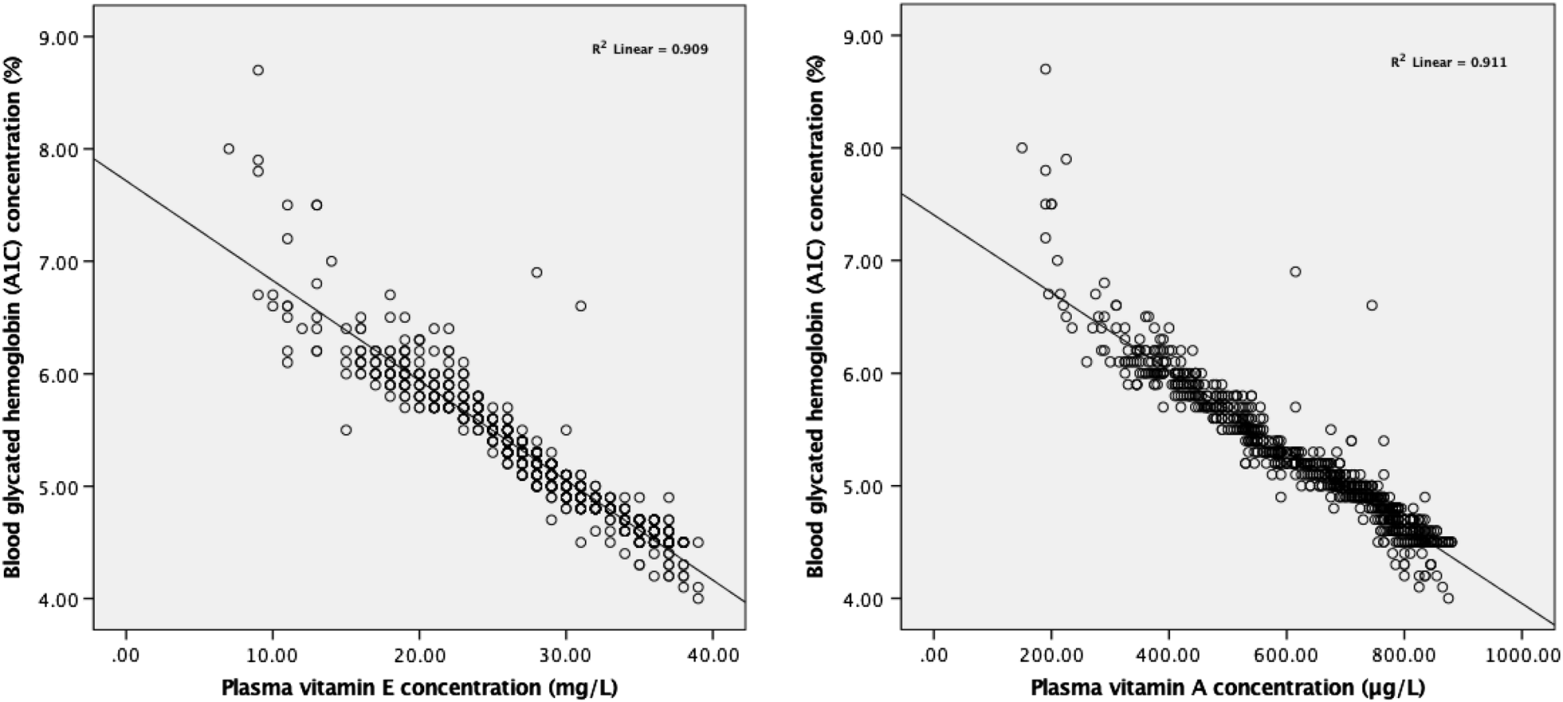
Linear regression analyses of A1C and vitamin A and E concentrations (n = 1102)

Linear regression analyses of A1C and plasma concentrations of vitamin A and E are illustrated in Fig. 3. Plasma concentrations of vitamin A and E were negatively associated with A1C concentration (B = − 003, 95% CI − 0.004 to − 0.003 and B = − 0.089, 95% CI − 0.090 to − 0.087, respectively), *P* < 0.001.

The multinomial regression analyses showed inverse associations between vitamin A and vitamin E concentrations and dysglycemia. Compared to normoglycemic status, the higher concentrations of vitamin A and E predicted lower odds of prediabetes (aOR = 0.951, 95% CI 0.94 to 0.96 and aOR = 0.272, 95% CI 0.21 to 0.35, respectively) and diabetes (aOR = 0.932, 95% CI 0.92 to 0.94 and aOR = 0.180, 95% CI 0.13 to 0.24, respectively) (Table 3).

**Table 3:** Multinomial logistic regression of the associations between vitamin A and E and A1C concentrations among 1st trimester pregnant Saudi women (n = 1102)

A1C^a^			B	SE	aOR	95% CI
Prediabetes						
Vitamin A (µg/L)						
Model 1			− 0.051	0.006	0.950^**^	0.94–0.96
Model 2			− 0.051	0.005	0.951^**^	0.94–0.96
Vitamin E (mg/L)						
Model 1			− 0.134	0.135	0.263^**^	0.20–0.34
Model 2			− 1.302	0.134	0.272^**^	0.21–0.35
Undiagnosed diabetes						
Vitamin A (µg/L)						
Model 1			− 0.072	0.006	0.930^**^	0.92–0.94
Model 2			− 0.071	0.006	0.932^**^	0.92–0.94
Vitamin E (mg/L)						
Model 1			− 1.776	0.154	0.169^**^	0.13–0.23
Model 2			− 1.715	0.153	0.180^**^	0.13–0.24

Model 1: unadjusted; Model 2: adjusted for age

*A1C* glycated hemoglobulin A1c, *B* beta, *SE* standard error, *aOR* adjusted odds ratio, *CI* confidence interval

^**^p < 0.001

^a^ The reference category is normal A1C concentration

## Discussion

In the present study, we aimed to evaluate the plasma concentrations of vitamin A and vitamin E and to examine the associations with the glycemic control status among first-trimester pregnant Saudi women. Analyses of data indicated inverse linear relationships between the plasma concentrations of vitamin A and E and A1C concentration. The plasma concentrations of vitamin A and E among the prediabetic participants were at a level midway between that of normoglycemic and diabetic participants. Compared to subjects with normoglycemic status, those with higher concentrations of vitamin A and E had lower odds of being prediabetic or diabetic. We are not aware of previous studies that have examined the antioxidant status among Saudi women with prediabetes or diabetes.

Emerging evidence proposed that oxidative stress has a key role in the etiology and pathophysiology of diabetes [15]. Chronic exposure of cells and tissues to hyperglycemia results in increased polyol pathway, enhanced protein glycosylation, and activates protein kinase C pathway. These altered mechanisms may trigger the production of reactive oxygen species in diabetic individuals [16, 17]. The role of reactive oxygen species in the pathogenesis of T2DM has been linked to the excessive oxidative stress and the antioxidant mechanism of diabetes [18]. The excessive production caused by hyperglycemia and/or the inadequate elimination of the reactive species cause oxidative stress through the generation of certain mechanisms that interfere with cellular physiological processes [19, 20]. On the other side, while the body has its own endogenous antioxidant systems and/or uses exogenous antioxidants from diet to neutralize the reactive oxygen species, any imbalance between the reactive species and the antioxidants may lead to overabundance of the reactive oxygen species and oxidative stress and, consequently, to the development of diabetes [15, 21].

In the present study, plasma concentrations of vitamin A and E among prediabetic and diabetic subjects were significantly lower than that of subjects with normoglycemic control; and levels of these vitamins in diabetic participants were significantly lower than that of prediabetic participants. This decrease could be due to increased oxidative stress associated with dysglycemia; It is also possible that the suboptimal intake of those antioxidant vitamins had contributed to the development of oxidative status and, consequently, to the initiation of prediabetes or the progression of prediabetes to diabetes [22]. Findings from other studies indicated that concentrations of vitamin A and E are possibly reduced due to the oxidative stress associated with dysglycemia and not a cause of this defect. For instance, a study compared the antioxidative status between 25 type-1 diabetic cases and 25 controls; serum vitamin A levels of diabetic cases were significantly lower than that of non-diabetic controls [23]. Another study conducted on 467 type-2 diabetic cases and 180 healthy controls. Lipid peroxidation was significantly raised within the first 2-years of diagnosis and vitamin E was significantly lower than that of non-diabetic subjects. These changes were correlated with the duration of the disease and were of a higher magnitude with the development of complications, suggesting that antioxidant deficiency and excessive peroxide-mediated damage may occur early before the development of T2DM complications [24]. Jovanovič et al. have also observed a significantly lower vitamin E concentration with increased T2DM duration compared to controls [25]. The reduced concentrations of vitamin A and E have been also observed among patients newly diagnosed with T2DM [26].

Several studies have observed declining concentrations of antioxidants among diabetic subjects; however, it is still not clear whether the reduced plasma concentration of antioxidants is the cause or the result of diabetes manifestation and data in this regard remain controversial [27]. In the present study, subjects with prediabetes had plasma concentrations of vitamin A and E at a level midway between normoglycemic and diabetic individuals, suggesting that the low concentrations of the studied nutrients may possibly have a contribution to the progression of prediabetes to diabetes. The low antioxidant concentrations among individuals with dysglycemia had been proposed in previous studies to be due to the low intake of these nutrients or the increased intake of antioxidants. A study compared dietary intake and serum concentrations of vitamin A and E between subjects with metabolic abnormalities and healthy subjects; lower consumptions have been observed among the cases. However, when the data were adjusted for the use of vitamins and minerals and intake of fruits and vegetables, the group with metabolic abnormalities still had lower serum concentrations of vitamin A and E, suggesting that the high levels of oxidative stress probably depleted endogenous and exogenous pools of antioxidants [28]. However, future prospective studies are needed to investigate the cause and effect relationships between the antioxidant status and hyperglycemia.

The therapeutic potential of antioxidant vitamins to alleviate the oxidative stress associated with T2DM have been previously investigated in clinical trials and positive outcomes were reported [29, 30]. In early pregnancy, antioxidant supplementation in women with low antioxidant status was found to improve maternal and perinatal outcomes and antioxidant status [31, 32]. On the other hand, excessive intake of vitamin A was found to increase risk for congenital malformations involving the central nervous and cardiovascular systems and spontaneous abortion [33, 34], while excessive intake of vitamin E was suggested to have an antagonistic effect on the absorption and functions of other fat-soluble vitamins [35]. A study conducted by Chen et al. investigated the serum concentrations of vitamin A and E across 28,023 samples of pregnant women; the data showed relatively low concentrations of vitamin A in early and late pregnancies; On the other hand, serum vitamin E concentrations were low in early pregnancy, while excess concentrations were observed in late pregnancies [36]. Therefore, proper assessment of the nutritional status should be mandatory in obstetric care; A special attention should be paid to pregnant women at risk of dysglycemia to evaluate their dietary intakes and risk of deficiencies. The clinicians should monitor the antioxidant status closely throughout pregnancy and provide nutritional guidance. Pregnant women whose vitamin A concentrations are low should be advised to consume animal products and deeply colored vegetables and fruits [37], whereas those with low vitamin E concentrations should be instructed on food sources rich in vitamin E, such as vegetable oils, nuts, and seeds [38]. Otherwise, antioxidant supplementation may be a rational treatment option to correct the nutritional deficiency.

Our study has several strengths. First, the sample size was large enough to improve the external validity of our findings. Second, this study was the first to investigate vitamin A and E concentrations of women with different glycemic control levels. Previous international studies have evaluated the antioxidant status among diabetic subjects, but data regarding vitamin A and E among prediabetic subjects are lacking. Therefore, our findings add to the evidence that lower concentrations of vitamin A and E are associated with the risk of T2DM. However, our study is limited by the nature of the cross-sectional study design. We were unable to confirm whether the abnormal levels of studied nutrients are the cause or the result of the prediabetes state. Future longitudinal studies could verify this relationship among the Saudi women. Additionally, we did not collect any dietary data. Even though previous studies confirmed that the state of dysglycemia is associated with low plasma concentrations of antioxidant vitamin levels, we were unable to determine whether the declined concentrations of vitamin A and E are due to the insufficient intake of food sources or due to changes in the metabolism of these nutrients. Conducting future research that investigate the association between dietary intakes and plasma concentrations of vitamin A and E might confirm whether this hypothesis is applicable to Saudi women.

## Conclusions

In the present study, negative linear relationships between plasma concentrations of vitamin A and E and A1C concentration were observed. Plasma concentrations of vitamin A and E in prediabetic women were at a level mid-way between normoglycemic and diabetic participants. Future studies are needed to elucidate the associations between the antioxidant status and dysglycemia. Clinicians should monitor the glycemic and antioxidant status closely and provide dietary guidance where needed.

## Data Availability

The data will be available from the authors upon reasonable request.
